# Feasibility of recruitment to an oral dysplasia trial in the United Kingdom

**DOI:** 10.1186/1758-3284-4-40

**Published:** 2012-06-25

**Authors:** Paul Nankivell, Janet Dunn, Michael Langman, Hisham Mehanna

**Affiliations:** 1 Institute of Head and Neck Studies and Education, University Hospitals Coventry and Warwickshire, Clifford Bridge Road, Coventry, CV2 2DX, UK; 2 Warwick Medical School, University of Warwick, Warwick, UK; 3 Clinical Trials Unit, University of Warwick, Warwick, UK; 4 University of Birmingham, Birmingham, UK

**Keywords:** Oral dysplasia, Aspirin, Clinical trial, Feasibility

## Abstract

**Background:**

Oral epithelial dysplasia (OED) has a malignant potential. Therapeutic options for OED remain both limited and without good evidence. Despite surgery being the most common method of treating OED, recurrence and potentially significant morbidity remain problematic. Consequently, there has been much interest in non-surgical treatments for OED. Cyclo-oxygenase (COX) up-regulation is known to occur in the dysplasia-carcinoma sequence and evidence now exists that COX-2 is a prognostic marker of malignant transformation in OED. COX-inhibitors are therefore considered a potential therapeutic strategy for treating this condition. We aimed to provide both proof of principal evidence supporting the effect of topical COX inhibition, and determine the feasibility of recruitment to an OED chemoprevention trial in the UK.

**Methods:**

Recruitment of 40 patients with oral leukoplakia to 4 study arms was planned. The total daily dose of Aspirin would increase in each group and be used in the period between initial diagnostic and follow-up biopsies.

**Results:**

During the 15-month recruitment period, 15/50 screened patients were eligible for recruitment, and 13 (87%) consented. Only 1 had OED diagnosed on biopsy. 16 patients were intolerant of, or already taking Aspirin and 16 patients required no biopsy. Initial recruitment was slow, as detection relied on clinicians identifying potentially eligible patients. Pre-screening new patient letters and directly contacting patients listed for biopsies improved screening of potentially eligible patients. However, as the incidence of OED was so low, it had little impact on trial recruitment. The trial was terminated, as recruitment was unlikely to be achieved in a single centre.

**Conclusion:**

This feasibility trial has demonstrated the low incidence of OED in the UK and the difficulties in conducting a study because of this. With an incidence of around 1.5/100,000/year and a high proportion of those patients already taking or intolerant of Aspirin, a large multi-centred trial would be required to fulfil the recruitment for this study. The ability of topical non-steroidal anti-inflammatory drugs to modify COX and prostaglandin expression remains an important but unanswered question. Collaboration with centres in other parts of the world with higher incidences of the disease may be required to ensure adequate recruitment.

**ISRCTN:**

31503555.

## Background

Oral epithelial dysplasia (OED) is a potentially malignant condition[1]. It often presents as oral leukoplakia, a characteristic white lesion of the oral mucosa. Between 5-46% of these lesions will have dysplasia on histological assessment[2-5]. OED has the potential to undergo malignant transformation to oral squamous cell carcinoma (OSCC). There is wide variability in reported transformation rates in the published literature from 5% to 36%[6,7]. Our recent meta-analysis estimated the malignant transformation rate to be around 12% (95% CI 8-18%)[8]. Despite 5-year survival rates improving slightly over the last 3 decades, progression to (OSCC) still carries a poor prognosis[9].

Therapeutic options for managing dysplasia in the oral cavity remain both limited and without a good evidence base. There are three principal treatment strategies; surveillance, chemoprevention and surgical[10]. Surgery is the most common method of treatment for dysplastic lesions, either by cold steel or LASER[11]. There are no randomized controlled trials (RCTs) directly comparing surgery to a surveillance policy, and there is no evidence to prove that surgery reduces the risk of malignant transformation in oral dysplasia[12]. Furthermore, recurrence rates after excision range between 10 and 35%. Patients who undergo repeated resections are exposed to potentially high levels of morbidity because of the anatomical areas involved[7,13,14]. Consequently, there has been an interest in non-surgical treatments for oral leukoplakia/dysplasia.

Clinical trials have examined a variety of chemoprevention strategies including the use of retinoids, bleomycin, carotenoids (beta-carotene and lycopene) tea, and systemic cyclo-oxygenase inhibitors. However, a Cochrane review of RCTs in oral leukoplakia failed to show any of them having a benefit over placebo in OED[12]. Despite this, there has been continued interest in the role of cyclo-oxygenase (COX) 1 and 2 and their inhibitors in oral premalignant lesions. COX 1/2 are known to be up-regulated in both oral dysplasia and cancer along with other malignancies of the gastrointestinal tract[15-18]. A study demonstrated a reduction in prostaglandin expression within the dysplastic lesions using systemic oral administration of a selective COX-2 inhibitor[19]. However, 2 separate trials of selective COX-2 inhibitors failed to demonstrate any significant effect in reducing the size or histological grade of oral dysplastic lesions. No biochemical effect was examined in these trials however[20,21]. Concerns over the cardiovascular side effect profile of systemic COX-2 inhibitors have limited further research in this area.

We wished to explore the efficacy of topical oral COX inhibitors on oral dysplasia. However, oral dysplasia is thought to be an uncommon condition in the UK[22,23]. Therefore, a clinical trial may be difficult to undertake in the UK. Our pilot study aimed to determine the feasibility of recruitment to an oral dysplasia chemoprevention trial in the UK, and to provide proof of principal evidence of the effect of oral topical COX inhibition on oral dysplastic lesions.

## Methods

Ethical approval from the West Midlands Research Ethics Committee (08/H1208/49) and a clinical trial authorisation from the MHRA were granted prior to the trial commencing. The trial was designed as a multi-centre, four-arm dose escalation, feasibility trial.

### Patients

Patients attending outpatient departments with a clinically diagnosed white patch requiring a biopsy for histological diagnosis were identified. Those meeting the inclusion and exclusion criteria outlined in Table 1 were approached for consent to participate in the trial.

**Table 1:** Patient eligibility criteria

** Inclusion criteria**	**Exclusion Criteria**
Clinically evident leukoplakia	Histologically confirmed cancer
Can attend for follow up	Prior oral cancer
Requiring biopsy	Patients on aspirin, non − steroidal anti-inflammatory drugs or corticosteroids
Over 18 years old	Current treatment of oral dysplasia involving topical or systemic treatment
Able to give informed consent	Active peptic ulcer
Not known to be pregnant	History of aspirin induced asthma, stomach ulcers or aspirin sensitivity
	History of associated angioedema or urticaria, or suspected oral allergic reactions in the past.

### Sample size

We intended to recruit forty patients in total, with ten patients sequentially allocated to each of the four study arms.

### Trial methodology

Consented patients underwent a biopsy of their lesion by a clinician or a research fellow. Half of the biopsy sample was sent for routine histological examination to provide a clinical diagnosis. The other half was immediately snap frozen to −80° C in liquid nitrogen for mRNA preservation. After histological assessment, those patients without a diagnosis of dysplasia on this first biopsy were reviewed in a follow-up clinic, and discharged from the study due to ineligibility. Those with a diagnosis of any grade of dysplasia were given a six-week course of Aspirin mouthwash and instructed on its use. Participants were asked to gargle with the mouthwash for at least one minute each time, two or three times daily as specified, and to expectorate it fully. They were also asked to record any adverse effects that they may notice during the period of using the mouthwash. Eligible patients would be sequentially allocated to one of the four dosing schedules. The total daily dose of Aspirin would increase sequentially in each group starting with 150 mg/day. The dosing schedules are shown in Table 2. Patients used the Aspirin for four to six weeks until they returned for an excision biopsy of the lesion. At this time, the excised tissue was treated in the same way as the initial biopsy tissue with half of the specimen used for routine histology to confirm the diagnosis and the other half for research purposes. Following excision, patients were followed up routinely in the head and neck clinic.

**Table 2:** Aspirin dosing schedule for each of the 4 study arms

**Group**	**Dosing schedule**	**Total Aspirin dose**
1	*One* dispersible 75 mg tablet in 250 ml of water used as mouthwash for 60 seconds *twice* a day.	150 mg
2	*Two* dispersible 75 mg tablet in 250 ml of water used as mouthwash for 60 seconds *twice* a day.	300 mg
3	*One* dispersible 300 mg tablet in 250 ml of water used as mouthwash for 60 seconds *twice* a day.	600 mg
4	*One* dispersible 300 mg tablet in 250 ml of water used as mouthwash for 60 seconds *three* times a day.	900 mg

### Outcome measures

The primary outcome measures were recruitment rates and Prostaglandin E2 levels by ELISA between the pre- and post-treatment biopsies as a measure of the activity of the oral topical COX inhibition. Secondary outcome measures included COX-1/COX-2 protein expression by immunocytochemistry and western blotting, the comparison of WHO histological grade, the clinical size of dysplastic lesion before and after treatment and the safety and tolerability of an Aspirin mouthwash, assessed by a patient diary.

### Analysis

Measurements outlined above were to be assessed on the initial diagnostic biopsy, and then repeated on the therapeutic excision specimen following 6 weeks of treatment. This would allow paired comparisons to be analysed using parametric and non-parametric methods, adjusting for dose of Aspirin as appropriate. Local and general tolerability along with side effects were assessed by a patient questionnaire.

## Results

### Screening

Initial detection of patients relied on clinicians identifying those that were potentially eligible and notifying the research team accordingly. Despite publicising the trial widely, only 3 patients were consented in the initial phase of the study. Two changes were therefore made which improved screening and consent rates. The first was pre-screening new patient letters and placing a reminder on the front of each set of notes. The second involved directly contacting those patients already listed for biopsies of the oral cavity. Despite improving screening of potentially eligible patients, as the rate of dysplasia was so low, it had little impact on trial recruitment.

### Consented patients

Recruitment took place at one site (University Hospital Coventry and Warwickshire NHS trust) between February 2010 and April 2011. During this period, 50 potentially eligible patients were screened. 15 were deemed eligible for recruitment, 13 (26%) of whom consented to participate in the trial. 2 patients declined to consent, citing a disinclination to participate in trials in general rather than any specific concerns regarding this trial protocol. Of the 13 consented, only 1 had dysplasia confirmed on their initial biopsy. The remaining 12 had diagnoses of lichen planus (2 cases), candidiasis (1 case), non-specific inflammation (8 cases) and one case of squamous cell carcinoma. The patient who entered the trial was allocated to trial group 1 and began the Aspirin mouthwash, but failed to attend 4 further follow up appointments, eventually returning after 4 months. As this patient had only used the mouthwash for 2 weeks, it was not felt to be beneficial to re-biopsy the lesion for analysis in the trial.

### Ineligible patients

For the 35 patients screened and found to be ineligible, the main reasons were that the patients were already on Aspirin (10 cases) were intolerant of Aspirin (6 cases) or the lesion was not felt to require a diagnostic biopsy (16 cases). The trial was terminated after 15 months as the trial steering committee considered recruitment of 40 patients was unlikely to be achieved with only a single centre recruiting. No other sites could be opened for recruitment due to the sponsor not wishing to sponsor an interventional drug trial at other sites.

### Missed patients

An audit was performed at the recruiting centre to assess the actual number of patients being diagnosed with oral dysplasia from the pathology database. 13 patients were diagnosed with dysplasia in our centre during the recruitment period. 5 were already taking or intolerant of Aspirin and 3 had a prior diagnosis of head and neck cancer. One patient was recruited in to the trial, with 4 potentially eligible patients missed. These patients all presented during the early phase of the trial, when recruitment relied on clinicians alerting the research team to potentially eligible patients. No patients were missed in the latter half of the trial period when the screening method was improved as discussed previously.

## Discussion

Failure of recruitment to this trial of topical COX-2 inhibitor for the treatment of OED is multifactorial. The main reason was low numbers of eligible patients, partly due to the low prevalence of the condition, but also to the high numbers of patients either already taking or intolerant of NSAIDs. Patient refusal did not appear to play a role. Some of these potential reasons are discussed in turn.

### Low incidence of oral dysplasia

There is little data on the incidence or prevalence of oral dysplasia. Following a workshop coordinated by the WHO collaborating centre for oral cancer and pre-cancer in the UK, a narrative review was produced examining the available epidemiological data[24]. The majority of this data has been derived from community-based surveys, predominantly from regions of rural India and from institutional reviews and case series from European and American centres. Nearly all primarily focus on the incidence and prevalence of leukoplakia and erythroplakia rather than specifically on the rates of dysplasia. This data can occasionally be extracted however.

Large cohort studies were carried out in India throughout the 1970’s. Two occupation specific studies (industrial workers in Gujarat and Bombay policemen) found annual incidence rates of 0.6 - 5.8 lesions/1000 per year amongst non-tobacco user. The incidence was much higher in tobacco users at 5.2 - 30.2/1000 per year[25,26]. Similar large studies in India have estimated the prevalence of leukoplakic lesions to be between 0.2% and 4.9%[27]. This difference in rates is thought to be because of regional variations in tobacco use. Slightly lower prevalence rates of 0.7% to 1.4% have been shown in European studies[28,29]. A study from 18 general dental practices in England examined 2265 patients over the age of 35. White or red lesions were found in 56 patients (2.5%)[30]. A systematic review by Petti attempted to pool the results from 23 published epidemiological studies. This analysis gave an estimated global prevalence of leukoplakia of 1.49 - 2.6% (95% CI 1.72 - 2.74) whilst acknowledging the high degree of heterogeneity between studies[31]. This heterogeneity is a key reason for the difficulty in calculating the true scale of the disease burden globally. In addition, many studies have a potential selection bias, as they are not representative of the overall population from which the cases are drawn.

The proportion of leukoplakic lesions harbouring dysplasia is also unclear. Published rates range from 5% to 46%[2-5]. In the population served locally (approximately 1 million people), an audit of dysplasia cases diagnosed over a one-year period has revealed 14 cases of oral dysplasia, giving an annual incidence of around 1.4 per 100,000. This rate appears similar to those found from other case series from the United Kingdom, where between 6–9 cases per year were diagnosed, (the size of the population these cases were drawn from is unclear, but all were tertiary referral centres)[22,23,32].

Our audit suggests that a typical Head and Neck unit in the UK may treat around one case of oral dysplasia per month (1 – 1.5 per 100,000 patient population per year) compared to the 10–15 Head and Neck Cancer cases per month. Oral dysplasia is therefore rarely seen in a teaching setting. This, combined with the ineligibility criteria, means any chance of recruitment would require large multi-centre trials in the UK.

### High ineligibility

A large proportion of patients were ineligible because they did not have a diagnosis of dysplasia, (27%) or did not undergo biopsy as there was not felt to be a clinical need to perform one (33%). A further 33% of potentially eligible patients could not be recruited, as they were either using, or intolerant of Aspirin. A change in trial design to allow previous or current use of systemic Aspirin may have improved that aspect of recruitment, but may also decrease the possibility of detecting an effect from oral Aspirin mouthwash.

### Incomplete patient capture

Continuous monitoring of patient recruitment is vital for clinical trial success. This surveillance allows interventions to be undertaken thereby maximising eligible patient enrolment. Slow initial recruitment in this study was identified by the use of screening logs. All clinic letters were then pre-screened to detect potentially eligible patients and highlight these to the relevant clinicians. This improved the numbers of patients consented. The most successful method of screening however was identifying patients on a waiting list for diagnostic biopsy. A dedicated list for outpatient biopsies further helped identification.

### Opening of other trial sites

Other sites for trial recruitment were identified. Despite willingness on the part of those centres, the local Research and Development department acting as trial sponsor declined to give approval for the trial to be opened at any other sites as this was the first drug trial to be sole-sponsored by them.

## Conclusion

This phase I feasibility trial has demonstrated difficulties in conducting a study on this condition in a single institution in the UK, probably due to the low incidence of oral dysplasia. With an incidence of only around 1 to 1.5 per 100,000 per year, and a high proportion of those patients already taking or intolerant of Aspirin, a large multi-centred trial would be required to fulfil the recruitment for this study. The question of the ability of topical non-steroidal anti-inflammatory drugs to modify COX and prostaglandin expression remains an important but unanswered question. Collaboration with centres in other parts of the world with higher incidences of the disease, such as India, may be a viable solution and is currently being undertaken by this group.

## Competing interest

The authors declare that they have no competing interests.

## Authors' contributions

PN, JD, ML and HM all contributed to the study design and methodology and write up of the manuscript. PN and HM were involved in the recruitment of patients and acquisition of data. JD contributed to the statistical aspects. All authors read and approved the final manuscript.
